# Evaluation of Stone Mastic Asphalt Containing Ceramic Waste Aggregate for Cooling Asphalt Pavement

**DOI:** 10.3390/ma13132964

**Published:** 2020-07-02

**Authors:** Qibo Huang, Zhendong Qian, Jing Hu, Dong Zheng

**Affiliations:** 1 Intelligent Transportation System Research Center, Southeast University, Nanjing 210096, China; hujing@seu.edu.cn (J.H.); dongzheng_seu@seu.edu.cn (D.Z.); 2 School of Transportation, Southeast University, Nanjing 210096, China

**Keywords:** cooling asphalt pavement, SMA, ceramic waste aggregate, road performance, thermal insulation performance, transient thermal field, rutting deformation

## Abstract

Construction and demolition waste material is of great potential for use in pavement engineering. This paper aims to investigate the feasibility of ceramic waste aggregate (CA) used in cooling asphalt pavement through a series of test methods and simulation techniques. Stone mastic asphalt (SMA) containing 10%, 20%, 30%, 40%, and 50% coarse ceramic waste aggregate (CASMAs) was first designed using the Marshall method. Afterward, the road performance and thermal insulation performance of the five different CASMAs were assessed by a comprehensive lab test, including a wheel rutting test, moisture susceptibility test, bending beam test, fatigue beam test, and indoor thermal insulation test. Finally, a 2D finite-element (FE) model was developed to investigate the transient thermal field and rutting deformation response of the cooling asphalt pavement with CASMAs. Results show that CASMAs experienced degradation of rutting resistance, moisture susceptibility, and anti-cracking performance while still meeting technical requirements with CA content of up to 40%. On the other hand, CASMAs can cool the pavement’s temperature by 11.5 °C at the bottom of asphalt layers. The permanent rutting deformation of cooling asphalt pavement was 45.36% smaller than that of conventional asphalt pavement without CASMAs. Based on the test results and numerical simulation results, the optimum content of ceramic waste aggregate in stone mastic asphalt was recommended as 40%.

## 1. Introduction

Asphalt pavement is one of the most widely applied transportation methods for passengers due to its high evenness, comfort, and low noise [1]. With the high solar heat absorptivity of asphalt, this black pavement usually suffers an extraordinary increase in structural temperature on hot days [2]. When subjected to both heavy traffic load and high temperature, this black pavement faces a high risk of rutting deformation because of the viscous flow of asphalt mixture. Moreover, asphalt pavement is liable to store and radiate a large amount of heat energy, leading to an exacerbation of the urban heat island (UHI) effect [3]. Therefore, there is a trend for urban traffic to require cooler and tougher asphalt pavement.

With the aim of dealing with the rutting problem of asphalt pavement, most efforts have been devoted to modifying the asphalt binder, adjusting aggregate gradation, and incorporating anti-rutting agents [4,5,6,7]. Some attention has also been paid to improving anti-rutting performance by lowering the structural temperature. Anting et al. [8] used three types of waste tile aggregate to develop a coating material for asphalt pavement. The surface temperature detected was performed at actual tropical weather climate and the results showed that a coating material with porcelain can obtain the largest surface temperature reduction of up to 6.4 °C. Synnefa et al. [9] conducted laboratory tests on the thermal performance of five thin colored layers of asphalt and performed a thermal fluid dynamics simulation of those samples on roads. It was found that a thin layer of colored asphalt can lower surface temperatures by as much as 10 °C compared with black asphalt. Jiang et al. [10] performed a series of tests to investigate the cooling effect, rutting resistance performance, and durability of thermally resistant course, heat-reflective coating, and thermally resistant and heat-reflective coating integrated pavement. It was concluded that thermally resistant and heat-reflective coating integrated pavement showed the best performance. Wang et al. [11] compared the cooling effect of different thermal-resistant aggregates on the asphalt mixture. Since the temperature of the thermal-resistant concrete drops rapidly over time, it was inferred that using thermal-resistant aggregate had an obvious cooling effect on the asphalt mixture.

On the other hand, the construction industry has constantly been producing large quantities of residue over the past decades [12,13]. Most of this residue is generated from construction and demolition, and mainly consists of concrete debris, cement mortar debris, and crushed ceramic [14,15]. Crushed ceramic is light in color and has a low thermal conductivity; it may help to reduce asphalt pavement’s storage of heat energy and lower the temperature of asphalt pavement. Therefore, the utilization of crushed ceramic waste in asphalt pavement is of great potential for controlling rutting defects, alleviating the UHI effect, and recycling solid waste.

Since using ceramic waste to replace natural aggregates may have considerable economic and environmental values, the limited research published has mainly focused on the feasibility of using ceramic waste in asphalt mixture. Hussein et al. [16] ground ceramic source waste to generate a nano ceramic powder (NCP) and investigated properties of bitumen modified by NCP. It was confirmed by the Asphalt Pavement Analyzer (APA) result that the rutting resistance of asphalt mixture was highly improved by the NCP. Muniandy et al. [17] conducted a comparative analysis of the chemical composition, particle size, morphology, and density of ceramic waste filler. It was concluded that the ceramic waste filler met the technical requirements for pavement use, and that asphalt mixture containing ceramic waste filler had better performance in rutting resistance compared to asphalt mixture with natural filler. Van De Ven et al. [18] analyzed the effect of coarse ceramic waste aggregate (CA) on the road performance of asphalt mixture. The test result showed that the addition of CA could improve the indirect tensile (IDT) strength, dynamic modulus, and fatigue life of asphalt mixture, but could impair the water stability of asphalt concrete. Ramón Silvestre et al. [19] concluded that using CA can increase the optimal asphalt content (OAC) and the air void of asphalt concrete, and asphalt mixture containing CA is recommended for roads with medium traffic. In summary, the evolution of performance during the service life of CASMAs has differences from that of conventional asphalt mixture, and little research has been carried out on both the cooling effect and the anti-rutting effect of CA on asphalt mixture. Therefore, further investigation of using ceramic waste aggregate in cooling asphalt pavement is still necessary.

## 2. Research Objective and Approach

**Figure 1:**
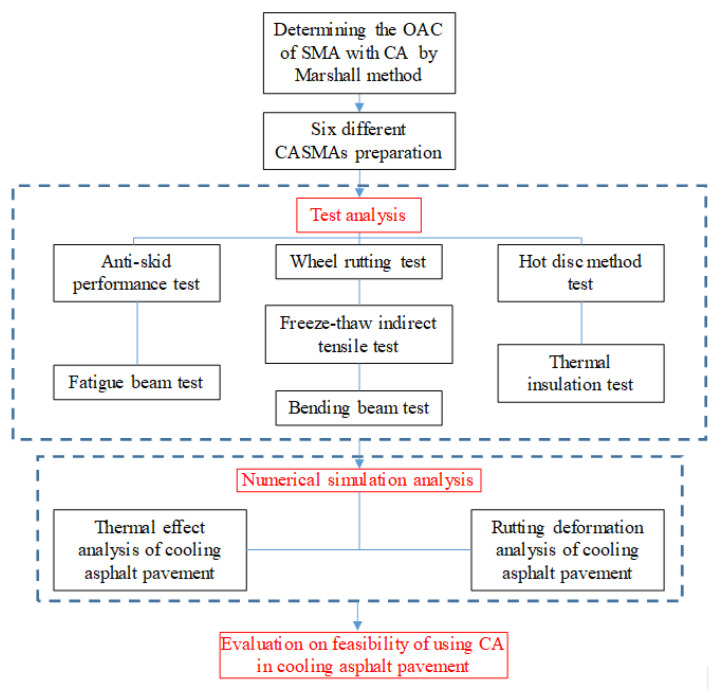
Flow chart of the research approach.

This paper mainly focuses on exploring the feasibility of ceramic waste aggregate used as a substitution for natural aggregate in asphalt mixture. Understanding the impact of ceramic waste aggregate on road performance and the thermal insulation characteristic of asphalt mixture can help to deal with rutting defects, ease the heat island effect, and consume solid waste. To that end, a series of tests and simulations were performed to evaluate the cooling effect and anti-rutting deformation performance of asphalt mixture containing ceramic waste. A flow chart of the research approach is shown in Figure 1.

## 3. Materials and Methods

### 3.1. Materials and Specimen Preparation

Stone mastic asphalt with gap gradation has a larger amount of coarse aggregate than conventional asphalt mixture with continuous gradation. More ceramic waste aggregate with small thermal conductivity can be used to replace natural coarse aggregates in stone mastic asphalt (SMA), which makes the thermal insulation performance of the asphalt mixture more significant. Therefore, this paper selected stone mastic asphalt (SMA) for the follow-up research.

#### 3.1.1. Aggregate and Filler

There are different types of ceramic waste, such as daily-use ceramics, bathroom ceramics, ceramic tiles, and insulating ceramics. The ceramic waste used in this paper was derived from waste ceramic tiles from the demolition of buildings, which belongs to the category of construction solid waste.

**Figure 2:**
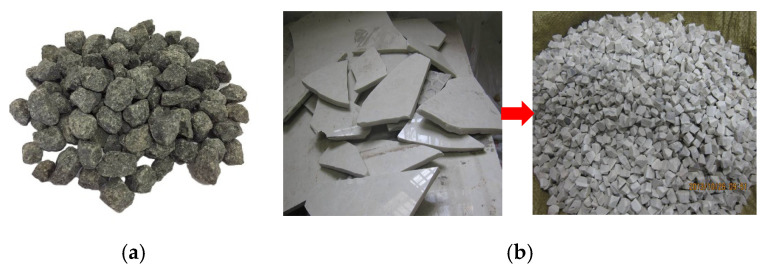
Aggregate used in this study: (**a**) Basalt aggregate; (**b**) Crushed ceramic waste aggregate.

The collected ceramic was washed, dried, crushed with a jaw crusher, sieved in a dry way, and stored in sizes of 5/10, 10/13, and 13/16 mm for the later preparation of the asphalt mixture. Natural basalt coarse aggregate was used as a control with a nominal maximum size of 13.2 mm. In addition, the fine aggregate used in the asphalt mixture was natural basalt, while the filler used was limestone. The fiber used in the stone mastic asphalt was lignin fiber. The morphologies of basalt aggregate and CA are shown in Figure 2.

The physical properties of aggregate and filler used are listed in Table 1. It was confirmed that the ceramic waste aggregate meet most specification values of the Superpave^TM^ Aggregate Specification Tests except for the index of flat and elongated particle content. As seen in Table 1, the density of ceramic waste aggregate is smaller than basalt, and this is mainly due to the large amount of micro-pore generated during the firing of ceramics. Aggregate with a porous structure can lower the heat absorption capacity of the asphalt mixture and reduce the heat transfer efficiency inside the pavement structure [11]. However, the crushing value and Los Angeles abrasion of ceramic waste aggregate (CA) are much higher than those of basalt aggregate. The main reason is that the ceramic tile is a more brittle material compared with natural aggregate, and it is easier to suffer brittle failure under the action of pressure and impact force.

#### 3.1.2. Asphalt Binder

To keep the stone mastic asphalt containing CA from cracking, rutting, and moisture damage, the asphalt binder used should have robust anti-aging performance, high viscosity, and good adhesion with the aggregate. The asphalt binder selected was Styrene butadiene styrene block polymer (SBS) modified asphalt, and its main technical indices are shown in Table 2.

#### 3.1.3. Mix Design

**Figure 3:**
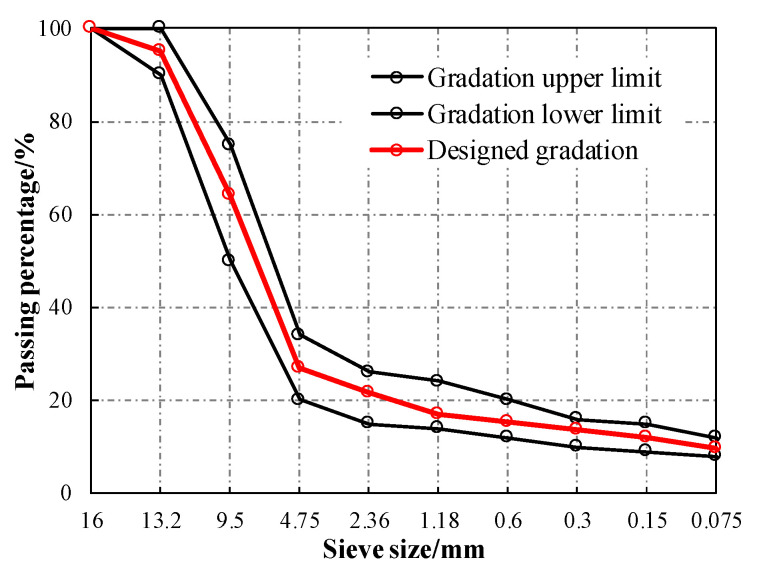
Designed gradation of CASMA-13 and SMA-13.

To ensure the comparability of test results, the gradations of stone mastic asphalt with coarse CA and coarse basalt aggregate were kept the same, and the synthetic gradation was selected according to JTG F40-2004 [33], as shown in Figure 3.

The particle sizes of ceramic waste aggregate were 5/10, 10/13, and 13/16 mm. Meanwhile, according to Figure 3, the volume ratios of basalt aggregates with particle sizes of 5/10 mm, 10/13 mm, and 13/16 mm were 37.2%, 31.1%, and 4.5%, respectively. As a result, specimens were prepared with different replacement percentages of ceramic waste aggregate (10%, 20%, 30%, 40%, and 50% by volume) blended with basalt aggregate. The aggregate was preheated at 190 °C, the mixing temperature of stone mastic asphalt was 175 °C, and the compaction temperature of stone mastic asphalt was 160 °C. Fifty blows of a Marshall hammer were applied on each side during the preparation of specimens.

With the incorporation of ceramic waste aggregate, the optimum asphalt content (OAC) of the stone mastic asphalt changed accordingly. Therefore, it was necessary to determine the OAC of stone mastic asphalt with different amounts of ceramic waste aggregate. According to JTG F40-2004, the OAC of SMA is determined using the air void acquired by the Marshall design method, and aggregate void in asphalt mixture filled with asphalt (VFA), void in mineral aggregate in asphalt mixture (VMA), Marshall stability, flow value, leakage loss value, and scattering loss values are used to validate the adequacy of SMA’s OAC. Based on a target porosity of 4.0%, the OACs of SMA-13, CASMA-13 (10%), CASMA-13 (20%), CASMA-13 (30%), CASMA-13 (40%), and CASMA-13 (50%) were determined as 5.7%, 5.8%. 5.9%, 6.0%, and 6.2%, respectively. The Marshall test results of stone mastic asphalt with optimum asphalt content are shown in Table 3.

The increasing OAC of CASMA-13 occurred mainly because ceramic waste aggregate has a higher water absorption than that of basalt aggregate. Secondly, the Marshall stability of stone mastic asphalt greatly decreased with the incorporation of ceramic waste aggregate, while the flow value increased. Moreover, the leakage loss value and scattering loss value of stone mastic asphalt both increased with the increase of ceramic waste aggregate. When the incorporation of ceramic waste aggregate was 50%, the leakage loss value exceeded the maximum specification value of 0.1%. This was mainly due to the poor adhesion of asphalt and ceramic waste aggregate, resulting in more free asphalt. In summary, the overall performance of stone mastic asphalt containing CA weakened as the ceramic waste aggregate increased, so the amount of CA in asphalt mixture should be limited.

### 3.2. Experimental Methods

Compared with conventional stone mastic asphalt (SMA-13), the road performance of CASMAs considering different ceramic waste aggregate content was assessed by carrying out an experimental program as follows: (a) a wheel rutting test to evaluate permanent deformation resistance; (b) a freeze-thaw indirect tensile test to assess moisture susceptibility at the freeze-thaw condition; (c) a three-point bending beam test to investigate resistance to cracking at low temperature; (d) a thermo-physical parameter test; (e) a mean texture depth (MTD) test and a British pendulum number (BPN) test to assess the skidding resistance of CASMAs; (f) a three-point fatigue beam test to study the fatigue resistance of CASMAs; (g) a thermal insulation test to investigate the insulation effect of CASMAs’ surface course. Table 4 lists the whole experimental program.

#### 3.2.1. Wheel Rutting Test

Slab specimens were manufactured by a roller compaction device. Aggregates and SBS modified asphalt were first mixed thoroughly at 175 °C. Then the filler was incorporated for the following mixing. Finally, mixtures were compacted under the roller at 160 °C to obtain a slab specimen (300 mm × 300 mm × 50 mm).

**Figure 4:**
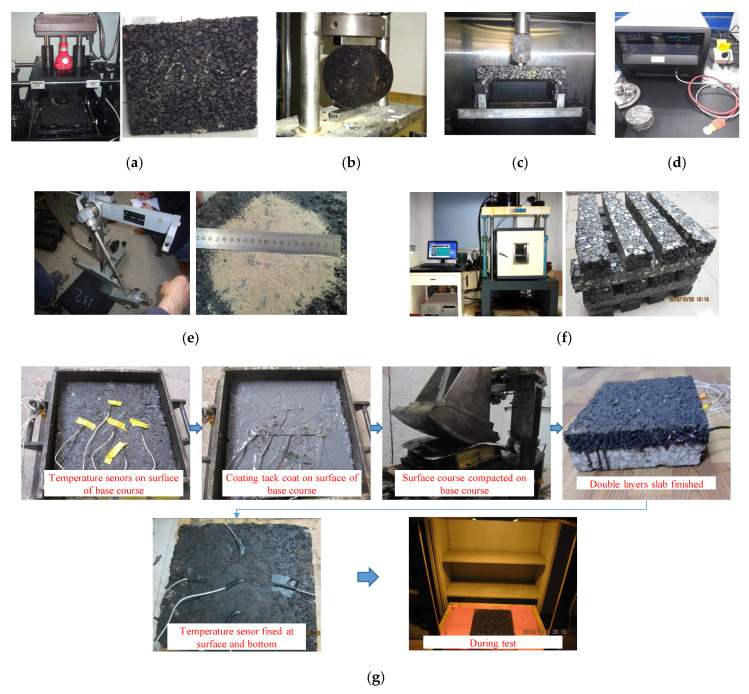
Test methods: (**a**) Wheel rutting test; (**b**) Freeze-thaw indirect tensile test; (**c**) Bending beam test; (**d**) HDM test; (**e**) Anti-skid performance test; (**f**) Fatigue beam test; (**g**) Thermal insulation performance test.

The wheel rutting test was carried out based on a procedure in the Chinese test specifications JTG E20-2011 T0719. During the test, a rubber wheel was subjected to the slab specimen, as presented in Figure 4a. The contact pressure was 0.70 MPa, and the temperature was 60 °C for the entire test. Linear variable differential transformers (LVDTs) were utilized to record the vertical deformation of the slab specimen during the test. Dynamic stability (DS) means the number of wheel passes required to beget one millimeter of rut deformation and can be calculated by Equation (1):

(1) $$DS=C_{1}\times C_{2}\frac{(t_{2}-t_{1})N}{d_{2}-d_{1}}$$

where *t*_1_ is 45 min and *t*_2_ is 60 min; *d*_1_ and *d*_2_ are the corresponding rut deformations; *C*_1_ and *C*_2_ are specific parameters of the wheel rutting tester and asphalt mixture type, here *C*_1_ = 1, *C*_2_ = 1; *N* is the passing speed of the rubber wheel, here *N* = 42 passes·min^−1^.

#### 3.2.2. Freeze-Thaw Indirect Tensile Test

A freeze-thaw indirect tensile test was conducted to assess the moisture susceptibility of CASMAs at freeze-thaw conditions, following a procedure in AASHTO T283. During the test, four Marshall specimens were placed in a 25 °C water bath for 2 h as an unconditioned group. The other four Marshall specimens were placed in a 25 °C water bath for 2 h, then conditioned for one freeze-thaw cycle. During the freeze-thaw cycle, the Marshall specimens were immersed in water under vacuum state for 15 min, then frozen at −18 °C in a refrigerator for 16 h, and then immersed in 60 °C water for 24 h. Finally, the indirect tensile strength (ITS) of the specimen was measured by a mechanical testing and simulation system (MTS) with a load rate of 50 mm/min at ambient temperature, as shown in Figure 4b. The tensile strength ratio (TSR) of CASMAs was defined by the ratio of ITS conditioned to unconditioned.

#### 3.2.3. Bending Beam Test

A three-point bending beam test was conducted to characterize the low-temperature cracking resistance of CASMA-13 following the procedure in JTG E20-2011 T0715. The rolling compacted slab specimen (300 mm × 300 mm × 50 mm) was cut into beam specimens with a size of 250 mm in length, 30 mm in width, and 35 mm in height. The beam specimens were then tested using a universal testing machine system (UTM-25, IPC, Melbourne, Victoria, Australia) with a load rate of 50 mm/min at −10 °C, as shown in Figure 4c.

#### 3.2.4. Hot Disc Method Test

CASMAs are expected to lower the temperature of the pavement structure. The thermo-physical parameters were measured by a hot disc method (HDM) test, according to the procedure in ASTM WK49591. The gyratory compacted cylinder specimen (Φ150 mm × H120 mm) was drilled and cut into a smaller cylinder specimen (Φ100 mm × H100 mm) to ensure that the surface was smooth enough for the hot disc method test. The test was performed on the cylinder specimen at 20 °C, and the external heat source for the specimen was simulated by the hot disc probe. In addition, the temperature evolution of specimen was measured by the hot disk probe, as shown in Figure 4d. The thermal conductivity, thermal diffusivity, and specific heat were calculated by the transient plane source method [39].

#### 3.2.5. Anti-Skid Performance Test

The anti-skid performance was assessed by indices of mean texture depth (MTD) and British pendulum number (BPN). The sand patch method test outlined in ASTM E965 was employed to measure the MTD, and the British pendulum test described in ASTM E303 was used to measure the BPN. Both of these two tests were carried out on the slab specimen (300 mm × 300 mm × 50 mm), as shown in Figure 4e.

#### 3.2.6. Fatigue Beam Test

Asphalt pavement is subjected to numerous repetitions of wheel loads and environmental conditions. Therefore, the materials used in asphalt pavement should have a great fatigue resistance performance to resist the generation of cracks in the pavement. In this study, the fatigue performance of CASMAs was assessed by a three-point bending beam test at 20 °C following the procedure in European Standard EN 12697-24, as shown in Figure 4f. The rolling compacted slab specimen was cut into beam specimens with a size of 300 mm in length, 40 mm in width, and 50 mm in height. Then the fatigue beam test was performed on these beam specimens. The load control mode with a half-sine waveform of 10 Hz frequency was selected to simulate the wheel load on the pavement, and the stress ratio was chosen as 0.6, 0.5, 0.4, and 0.3 [40]. In this paper, the fatigue life of stone mastic asphalt was defined as the number of load cycles required for a 50% reduction of the beam specimen’s initial stiffness.

#### 3.2.7. Thermal Insulation Test

**Figure 5:**
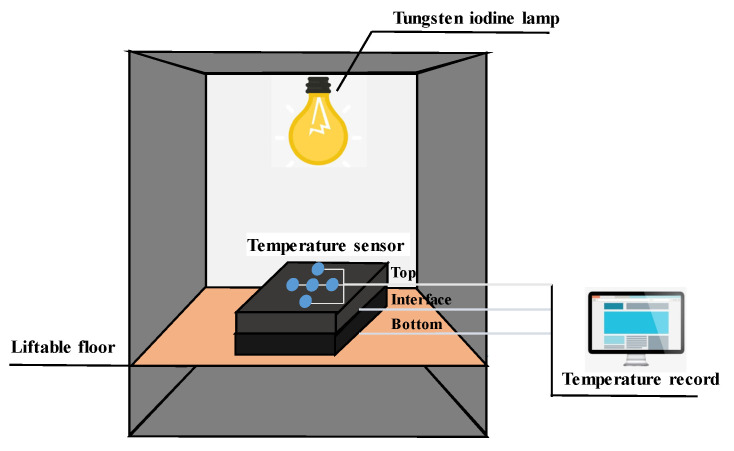
Schematic diagram of sunshine simulator.

An indoor thermal insulation test was designed as shown in Figure 5. A tungsten iodine lamp with a power of 500 W was used to simulate solar radiation.

A double-layer slab specimen with a 4 cm surface course and a 6 cm base course (AC-20) was fabricated, and the temperatures of the surface, interface, and bottom were recorded through a thermocouple thermometer. The AC-20 mixture with a continuous gradation was made of limestone coarse aggregate, basalt fine aggregate, limestone filler, and SBS modified asphalt. Figure 4g shows the fabrication procedure of the composite specimen. The height of the liftable floor was adjusted to ensure that the lamp’s illuminance on the specimen’s surface was 140,000 lx [41]. Conventional stone mastic asphalt (SMA-13) was used as a control group to compare the thermal insulation effect of CASMAs. Additionally, the composite specimen was under radiant heating for 8 h to ensure a stable temperature within the specimen.

### 3.3. Numerical Model

To assess the influence of CASMAs on the thermal field and the rutting resistance of cooling asphalt pavement, a finite element model was established to analyze the temperature changing law of asphalt pavement structure with CASMAs. Based on the result, further simulation of permanent deformation under the impact of thermal field and wheel load was conducted.

#### 3.3.1. Theoretical Background

Due to exposure to the outdoor environment, the temperature distribution of asphalt pavement presents continuous spatiotemporal variation with periodic climatic conditions. During this variation, the heat is conducted through the surface course, road base, and subgrade, leading to a complex and changeable thermal field. Therefore, the thermal field of the asphalt pavement structure under outdoor environmental conditions should be regarded as a transient thermal field. As for isotropic homogeneous materials, the unsteady heat conduction in the three-dimensional space follows the Fourier partial differential equation and energy conservation law [42]. In other words, the transient thermal field of asphalt pavement is governed by the differential equation as follows:

(2) $$\rho \cdot c\frac{\partial T}{\partial t}-\lambda \cdot \left(\frac{\partial^{2}T}{\partial x^{2}}+\frac{\partial^{2}T}{\partial y^{2}}+\frac{\partial^{2}T}{\partial z^{2}}\right)-Q=0$$

where *ρ* is the density of pavement material (kg/m^3^); *c* is the specific heat of pavement material (J·kg^−1^·K^−1^); *λ* is the thermal conductivity of pavement material (W·m^−1^·K^−1^); *x*, *y*, and *z* are Cartesian coordinates; and *Q* is the heat generated internally per unit volume, here *Q* = 0.

Combined with the boundary condition of heat conduction, the temperature distribution of asphalt pavement can be obtained following the differential Equation (2). Based on the theory of heat conduction, the thermodynamic boundary conditions of pavement can mainly be classified into two types. Once the temperature of the pavement structure boundary is designated, the thermodynamic boundary condition can be regarded as the first type, namely the Dirichlet condition, which is defined as:

(3) $$T\left(x,y,z,t\right)=\bar{T}\left(t\right)$$

where $\bar{T}$*(t)* is the designated temperature of the pavement structure boundary (°C).

**Figure 6:**
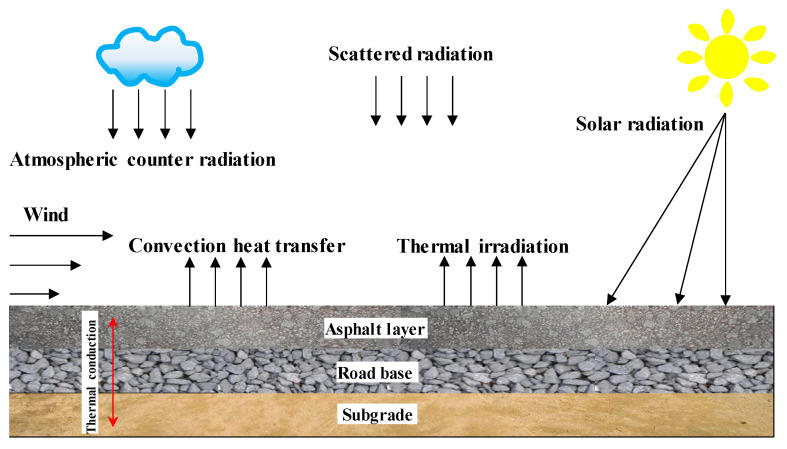
Environmental conditions of asphalt pavement structure.

Once the heat flux density at the boundary of the pavement structure is prescribed, the thermodynamic boundary condition can be regarded as the second type, namely the Neumann condition, which is defined as:

(4) $$\lambda \left(\frac{\partial T}{\partial x}n_{x}+\frac{\partial T}{\partial y}n_{y}+\frac{\partial T}{\partial z}n_{z}\right)=\bar{q}\left(t\right)$$

where *n_x_*, *n_y_*, and *n_z_* are direction cosines of the outward normal to the pavement surfaces, respectively, and $\bar{q}$*(t)* is the prescribed heat flux density at the pavement structure boundary (W/m^2^). As for asphalt pavement exposed to the outdoor environment, the heat flux density $\bar{q}$*(t)* at the pavement surface includes solar radiation *q_s_*, convection heat transfer *q_c_*, and thermal irradiation *q_r_* [43], as presented in Figure 6.

Solar radiation consists of scattered radiation and direct solar radiation, and the daily variation of solar radiation *q_s_* can be calculated following Equation (5) [44]:

(5) $$q_{s}=\{\begin{array}{cc} 0, & 0\leq t<12-c/2\\ q_{0}\cos m\omega (t-12), & 12-c/2\leq t\leq 12+c/2\\ 0, & 12+c/2<t\leq 24 \end{array}$$

where *q*_0_ is the maximum solar radiation at noon, *q*_0_ = 0.131 m *Q*; *Q* is the total daily solar radiation (J/m^2^); *c* is the daily effective sunshine hours (h); *m* is the distribution coefficient for solar radiation, *m* = 12/*c*; *ω* is the angular frequency, *ω* = 2π/24 (rad).

During convective heat transfer, heat exchange occurs between the asphalt pavement surface and adjacent air. Based on Newton’s convection cooling law [45], the convection heat transfer *q_c_* can be calculated as:

(6) $$q_{c}=\beta \cdot A\cdot (T-T_{a})$$

where *β* is the convection heat transfer coefficient (W·m^−2^·K^−1^); *β*= 3.0*v* + 5.6, in which *v* is the wind speed (m·s^−1^); A is the contact area (m^2^); and *T_a_* is the air temperature (°C).

While absorbing the radiation emitted from the environment, the asphalt pavement also radiates energy in the form of long-wave radiation. According to the Stefan–Boltzmann law [43], the thermal irradiation *q_r_* between the pavement surface and adjacent air can be defined by using the following formula:

(7) $$q_{r}=C_{s}\times e\times \left[{\left(T-T^{*}\right)}^{4}-(T_{a}-T^{*})\right]$$

where *e* is the emissivity coefficient of the asphalt pavement surface, here *e* = 0.81; *C_s_* is the Stefan–Boltzmann constant, *C_s_* = 5.6697 × 10^−8^ W m^−2^ K^−4^; and T* is the absolute zero degree, T* = −273 °C.

Combined with Equations (5)–(7), the temperature boundary condition of the asphalt pavement could be calculated according to Equation (4):

(8) $$\lambda \left(\frac{\partial T}{\partial x}n_{x}+\frac{\partial T}{\partial y}n_{y}+\frac{\partial T}{\partial z}n_{z}\right)=q_{s}+\beta \cdot A\cdot (T-T_{a})+C_{s}\times e\times \left[{\left(T+T^{*}\right)}^{4}-(T_{a}+T^{*})\right]$$

#### 3.3.2. FE Model for Pavement Thermal Field and Mechanical Response Analysis

**Figure 7:**
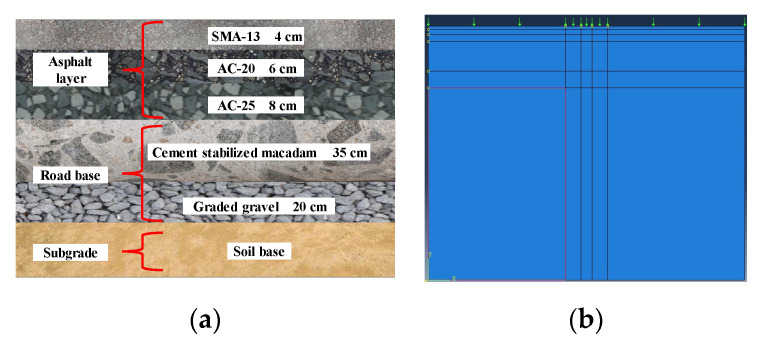
Ordinary asphalt pavement structure in China: (**a**) Asphalt pavement structure; (**b**) 2D FE model.

An ordinary urban asphalt pavement structure used in Kunshan city in Jiangsu province was considered in this study, as shown in Figure 7a. The asphalt layers consisted of a surface course (4 cm SMA-13), an intermediate course (6 cm AC-20), and a base course (8 cm AC-25). A drilled specimen from each asphalt layer was tested for thermophysical properties through the hot disc method test. In contrast, the thermophysical properties of the road base and subgrade were obtained from the literature [46]. Here, road base consisted of a cement stabilized macadam layer (35 cm CSM) and graded gravel (20 cm GG). The corresponding thermal parameters are listed in Table 5.

The time hardening creep model was applied to describe the viscoelastic behavior of asphalt mixture under repeated wheel load. The time hardening creep model is shown in Table 6.

The model parameters of *A*, *n*, and *m* for asphalt mixture were used as inputs in the FE model. Because of the temperature dependency of asphalt mixture, the tri-axial repeated loading creep test and compressive rebound modulus test were both conducted at 20, 30, 40, 50, and 60 °C, respectively. On the other hand, the materials used in the road base and subgrade were regarded as linear elastic. The viscoelastic parameters of SMA and CASMAs are listed in Table 6 as an example.

A two-dimensional finite element model created by the finite element software ABAQUS 6.11 was used to calculate the rutting deformation of asphalt pavement under the impacts of repeated wheel load and thermal field. Figure 7b shows the 2D FE model of the pavement structure. In the model, all the layers were modeled by the shell element, and the tied contact was set between the layers assuming that the deformation of different layers was continuous. The boundary condition of the model was constrained for deformation in all directions. According to the analysis of size sensitivity, the width and depth of the asphalt pavement model were set 3.75 and 3 m, respectively. The transient thermal field of asphalt pavement was first simulated considering solar radiation, convective heat transfer, thermal irradiation, and conduction between pavement layers. Based on the temperature field of asphalt pavement, the stone mastic asphalt’s creep parameters in the 2D FE model were modified, and cumulative rutting deformation of asphalt pavement caused by the repeated wheel load was then calculated.

The applied wheel load was a 100 kN dual-tire assembly with a speed of 60 km/h. Constant static load was applied in the 2D FE model to simulate the repeated dynamic wheel load. Thus, the corresponding number of passes of the repeated dynamic load could be converted into the cumulative time of constant static load. The tire–pavement contact stress was simplified as an equivalent dual-rectangular uniform pressure of 0.7 MPa. More details on constant static load and dual-rectangular uniform stress can be found in one of the authors’ previous publications [47].

#### 3.3.3. Model Verification

**Figure 8:**
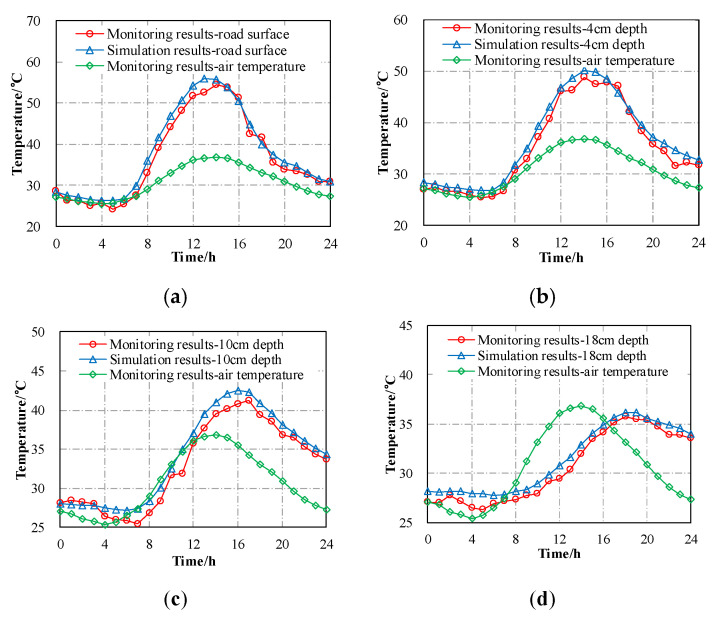
Simulation results compared with the monitoring results: (**a**) Road surface; (**b**) 4 cm depth; (**c**) 10 cm depth; (**d**) 18 cm depth.

A field experiment was carried out to monitor the thermal field of asphalt layers in pavement structure through embedded thermocouples. The total daily solar radiation measured was 22.3 × 10^6^ J/m^2^, the average wind speed was 2.4 m·s^−1^, and the daily effective sunshine was 11.3 h. The monitoring and simulated temperatures of asphalt pavement at different depths are shown in Figure 8.

It can be found from Figure 8 that most of the simulated pavement temperatures were higher than that of the monitoring values, which means that the calculation from the FE model was conservative. This observation is because the heat dissipated caused by water evaporation was not considered in the FE model. On the other hand, the maximum temperature difference between the calculated value and the monitoring value was 3.4 °C, and the corresponding error was about 6.9%, which indicates that the 2D FE model in this study could be used to predict the thermal field of the asphalt pavement structure.

**Figure 9:**
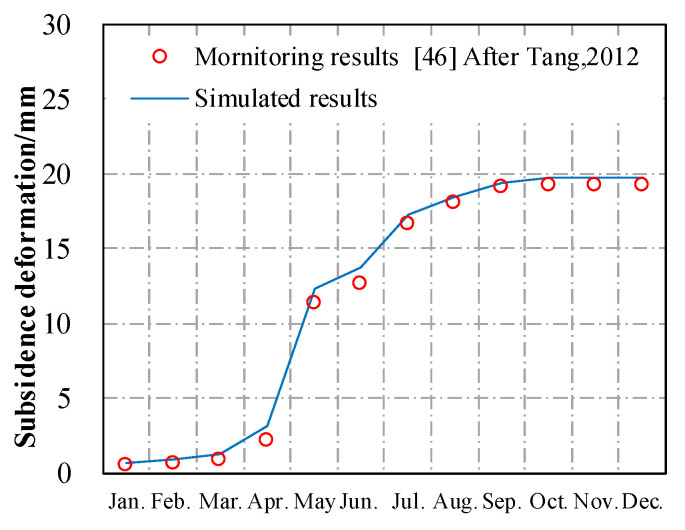
Monitoring and simulated cumulative rutting deformation subsidence.

The rutting deformation of the asphalt pavement in the reference was calculated through the parameters recorded [46]. The subsidence part of the simulated cumulative rutting deformation was compared with the instrumentation measurements in the experimental pavement, as presented in Figure 9. The result shows that the simulated rutting deformation matches well with field data recorded in the reference, which indicates that this FE model is reliable for the deformation analysis of asphalt pavement structure.

## 4. Results and Discussion

### 4.1. Experimental Results

Implemented as the surface course of cooling asphalt pavement, CASMAs should not only meet the conventional technical requirements of asphalt pavement but also have good thermal insulation performance. Table 7 summarizes the test results of CASMAs and SMA.

As shown in Table 7, the dynamic stability of the stone mastic asphalt decreased as the content of ceramic waste aggregate increased, indicating that the more CA, the more significant rutting deformation of the stone mastic asphalt. Additionally, the largest decreasing amplitude of dynamic stability for the stone mastic asphalt was found within a CA content range of 40% to 50%. The lowest dynamic stability value of CASMAs was 62% smaller than that of SMA-13, which failed to meet the technical requirement of over 3000 pass·mm^−1^. This phenomenon may be attributed to the higher crushing value and flat and elongated particle content for ceramic waste aggregate. Therefore, the content of ceramic waste aggregate used in stone mastic asphalt should not exceed 40% in terms of rutting resistance.

As for moisture susceptibility, the TSRs of all stone mastic asphalt, except for CASMA-13 containing 50% CA, met the criteria requirement (≥75%). The worse TSR of CASMAs was about 15% smaller than that of SMA. This phenomenon is mainly due to the poor adhesion between ceramic waste aggregate and asphalt. It could be concluded that increasing the CA content goes against the freeze-thaw moisture damage resistance of stone mastic asphalt.

Failure bending strain is an important index for evaluating the cracking resistance performance of stone mastic asphalt. As found, the maximum bending strain of SMA-13 was higher than that of CASMAs. The smallest bending strain of CASMAs (for the case of 50% CA) was 441 με lower than that of SMA-13. In the three-point bending beam test, the bending strength of CASMAs ranged from 11.73 to 12.86 MPa, while the bending strength of SMA-13 was 13.26 MPa. All these observations indicate that the cracking resistance performance of stone mastic asphalt decreases with the increase of CA content.

With the increase of ceramic waste aggregate in stone mastic asphalt, thermal conductivity and thermal diffusivity gradually decreased, while the specific heat increased. Incorporation of 10% CA into stone mastic asphalt could result in a 23.5% reduction of thermal conductivity, a 20.9% reduction of thermal diffusivity, and a 20.9% increment of specific heat. On the other hand, the incorporation of 50% CA into stone mastic asphalt made little difference compared with that of 40% CA, revealing that the improving effect of ceramic waste aggregate on thermal insulation slowed down once the content exceeded 40%. As a whole, it could be inferred that CASMAs can be an attractive material for the thermal insulation of asphalt pavement.

The results of the BPN test and MTD test illustrate that the conventional SMA-13 and the stone mastic asphalt containing CA can both meet the criteria of SMA’s skid resistance, and could provide friction as a surface course of asphalt pavement.

**Figure 10:**
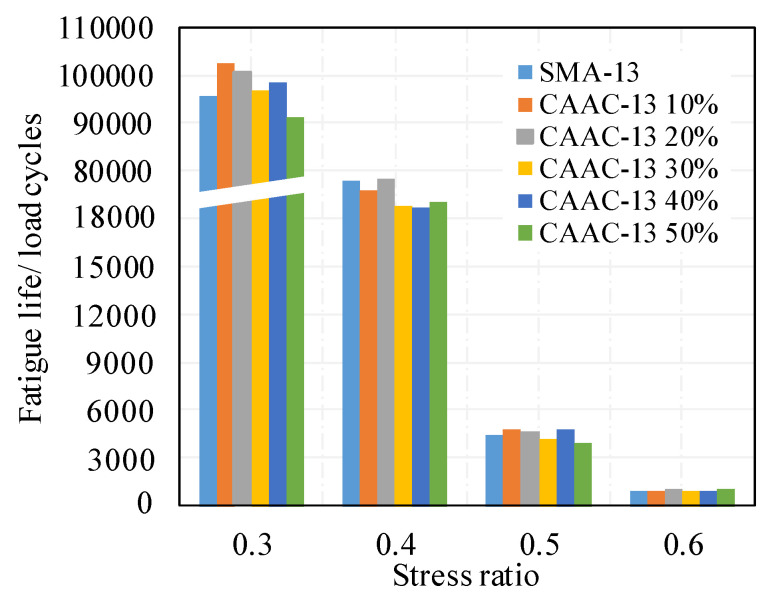
Fatigue life of beams at different stress ratios.

Figure 10 presents the fatigue resistance performance of stone mastic asphalt with different contents of ceramic waste aggregate. As the stress ratio increased from 0.3 to 0.6, the fatigue life of stone mastic asphalt decreased sharply. When subjected to the same stress ratio, the tested fatigue life of SMA-13 had no obvious difference from that of the CASMAs, revealing that the incorporation of ceramic waste aggregate may not harm the fatigue resistance performance of stone mastic asphalt.

Two-way ANOVA was conducted for statistical analysis of the fatigue resistance performance of stone mastic asphalt with different CA contents at a 95% confidence level. The results are listed in Table 8. Various stress ratios significantly affect fatigue life in the statistics, but the contents of ceramic waste aggregate have no significant effect on fatigue life in the statistics. Therefore, it could be concluded that CASMAs could meet the requirements for resisting fatigue damage at mid-temperature.

The temperatures at the surface, interface, and bottom of the double-layer slab specimen (CASMA-13 + AC-20) were recorded during the indoor thermal insulation performance test, and the results are shown in Table 9.

It can be found from Table 9 that the surface temperature gradually increased with the increase of ceramic waste aggregate in CASMAs. This phenomenon is due to the more significant heat absorption of CASMA-13 caused by higher optimal asphalt content. In contrast, the interface temperature and bottom temperature both decreased as the ceramic waste aggregate increased. Simultaneously, the surface–interface temperature gap and surface–bottom temperature gap increased. This was because the incorporation of ceramic waste aggregate could lower the thermal conductivity and thermal diffusivity of the surface course, and helped to weaken the heat conduction between layers. For example, the surface–interface temperature gap of CASMA-13 (50% CA) + AC-20 was 8.8 °C larger than that of SMA-13 + AC-20. Similarly, the surface–bottom temperature gap of CASMA-13 (50% CA) +AC-20 was 11.5 °C larger than that of SMA-13 + AC-20. Although the temperature gap here maybe deviated from the field’s actual situation, it could be inferred that asphalt pavement with a CASMAs surface course has better performance in terms of the cooling effect than that with conventional SMA.

### 4.2. Numerical Results

#### 4.2.1. Thermal Effect Analysis of Cooling Asphalt Pavement

**Figure 11:**
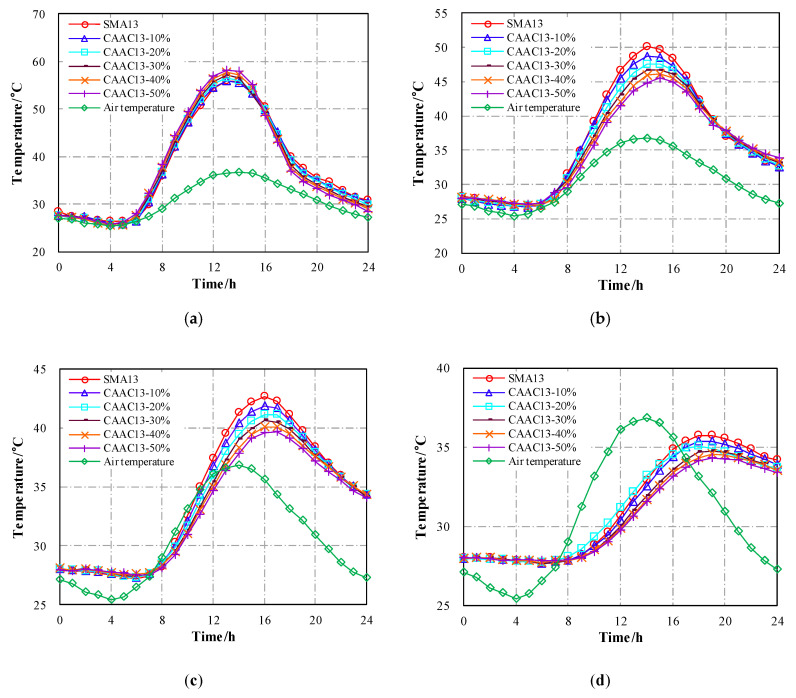
Effect of CASMA-13 on the temperature evolution of asphalt layers: (**a**) Road surface; (**b**) 4 cm depth; (**c**) 10 cm depth; (**d**) 18 cm depth.

Based on the validated thermal field model, the thermophysical inputs of surface course material (CASMAs) were modified to analyze the temperature distributions of cooling asphalt pavement with different CA contents. The thermal simulation results for asphalt layers in the cooling asphalt pavement are given in Figure 11.

When CASMAs were implemented as a surface course of the pavement structure, the maximum temperature of the road surface increased with the increase of CA content, while the maximum temperatures within the asphalt pavement gradually decreased with the rise of CA content. The reason might be that ceramic waste aggregate could reduce thermal conductivity, resulting in poorer heat conduction between the cooling asphalt pavement layers. More heat was accumulated within the surface course, and the amount of heat transferred downward become smaller. Thus, temperatures at the intermediate and base course of asphalt layers dropped. Taking the cooling asphalt pavement containing CASMA-13 (50% CA) as an example, the maximum temperatures at depths of 4 cm, 10 cm, and 18 cm were 5.5 °C, 3.2 °C, and 1.6 °C lower than that of conventional asphalt pavement without a CASMA thermal insulation layer. On the other hand, the amplitude of thermal conductivity reduction got smaller when the incorporation of ceramic waste aggregate exceeded 40%, accompanied by a smaller amplitude of temperature reduction within the asphalt pavement, as shown in Figure 11. Combining the test results of road performance and the simulation results for temperature reduction, the content of ceramic waste aggregate used in stone mastic asphalt was recommended as 40%. As a result, the rutting deformation of the cooling pavement including a CASMA-13 (40% CA) surface course was further investigated and is described in the next section.

#### 4.2.2. Rutting Deformation Analysis of Cooling Asphalt Pavement

**Figure 12:**
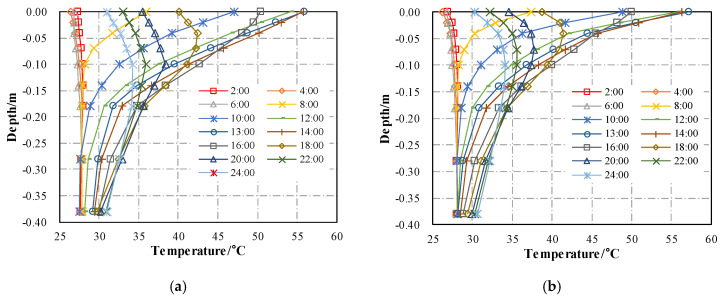
Asphalt pavement temperature and depth at different times: (**a**) Conventional asphalt pavement; (**b**) Cooling asphalt pavement with CASMA thermal insulation layer.

The viscoelasticity of asphalt mixture is very sensitive to temperature. It is necessary to introduce the initial thermal field into each analysis step when simulating the rutting deformation of asphalt pavement. The simulated temperature distributions of cooling asphalt pavement and conventional asphalt pavement at different times are shown in Figure 12.

**Figure 13:**
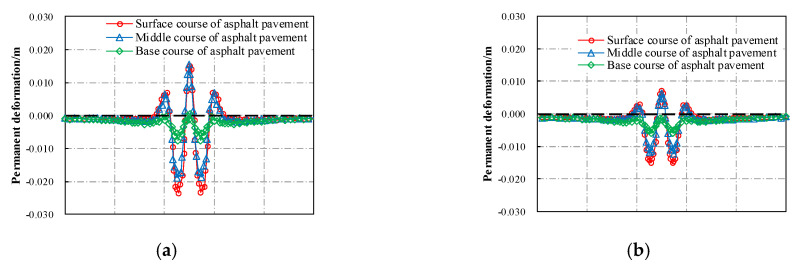
Distributions of permanent deformation at different depths: (**a**) Conventional asphalt pavement; (**b**) Cooling asphalt pavement with CASMA thermal insulation layer.

**Figure 14:**
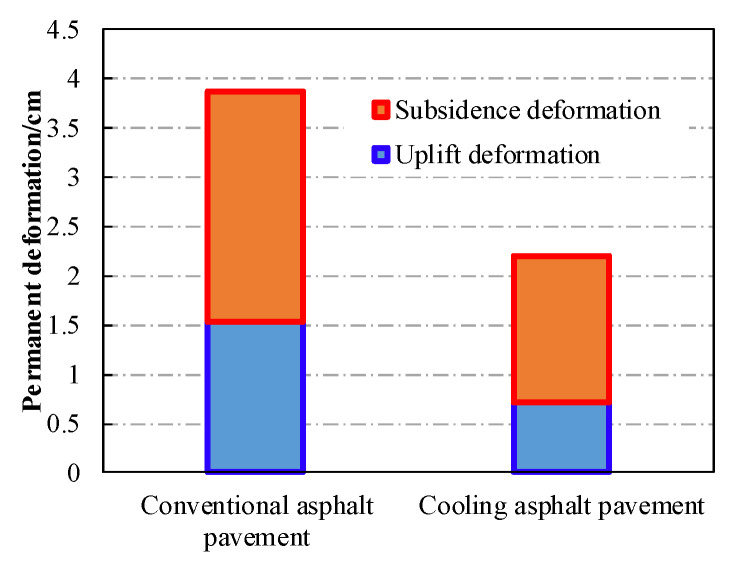
Rutting deformations of two different asphalt pavement structures.

Based on the validated FE model, the rutting deformations of different pavement structures under repeated wheel load and environmental impact were analyzed. Conventional pavement structure (without a thermal insulation layer) was used as a reference group for the cooling asphalt pavement (with a CASMA thermal insulation layer). The corresponding simulated results of permanent deformation after 500,000 passes of axle loads are shown in Figure 13 and Figure 14.

When subjected to long-term repeated wheel load, each asphalt layer of the pavement suffered a visible rutting deformation. The maximum subsidence deformation occurred right below the center of the wheel load. Furthermore, uplift deformation also appeared adjacent to the wheel load due to the lateral flow of asphalt mixture. Since the conventional asphalt pavement absorbed more heat and transferred it faster to the lower layers, the temperature within this pavement was higher than that of the cooling asphalt pavement with the CASMA thermal insulation layer. A higher temperature could lead to a worse rutting resistance of asphalt mixture. As a result, the permanent rutting deformation of the cooling asphalt pavement (2.12 cm) was much smaller than that of conventional asphalt pavement (3.88 cm), and the corresponding reduction of rutting deformation was 45.36%. Although the simulated rutting deformation here maybe deviated from the field’s actual situation, it could be inferred that the cooling asphalt pavement with a CASMA thermal insulation layer had better performance in rutting resistance than that of the conventional structure.

## 5. Conclusions

This paper investigated the feasibility of utilizing ceramic waste as the coarse aggregate in cooling asphalt pavement. Stone mastic asphalt (SMA) containing 10%, 20%, 30%, 40%, and 50% coarse ceramic waste aggregate (CASMAs) was first designed using the Marshall method. Afterward, the road performance and thermal insulation performance of the five different CASMAs were assessed by a comprehensive lab test, including a wheel rutting test, moisture susceptibility test, bending beam test, fatigue beam test, and indoor thermal insulation test. Finally, a 2D finite-element model was developed to investigate the transient thermal field and rutting deformation response of the cooling asphalt pavement with CASMAs. The following conclusions can be drawn based on the analysis:

(1) As the incorporation of ceramic waste aggregate increased, the stone mastic asphalt’s optimum asphalt content and flow value gradually increased, while the stone mastic asphalt’s Marshall stability and density gradually decreased.
(2) The CASMAs designed in this paper experienced degradation of some road performances as the content of ceramic waste aggregate increased. The smallest dynamic stability of CASMAs was about 62% lower than that of conventional SMA. The smallest TSR value and failure bending strain of CASMAs were about 15% smaller than those of SMA. In contrast, SMA and CASMAs have no significant difference in fatigue life, and both can provide friction as a surface course of cooling asphalt pavement.
(3) The thermal conductivity of conventional SMA was 1.25–2.28 times that of CASMAs, and CASMAs could be an attractive material for the thermal insulation layer of cooling asphalt pavement. The indoor thermal insulation analysis confirmed that CASMAs had better thermal insulation performance than SMA. The best cooling effect was an 8.8 °C reduction in temperature at the interface, and an 11.5 °C reduction in temperature at the bottom, respectively.
(4) When CASMAs were implemented as a surface course in the thermal field simulation model, the pavement surface temperature increased, while the temperature within the pavement decreased. Combining the test results of road performance and thermal simulation results, the optimal content of ceramic waste aggregate in stone mastic asphalt was recommended as 40%.
(5) When subjected to repeated traffic load outdoors, the permanent rutting deformation of cooling asphalt pavement was 45.36% smaller than that of conventional asphalt pavement, indicating that the cooling asphalt pavement with CASMA thermal insulation layer had a better anti-rutting performance.

## Figures and Tables

**Table 1:** Physical properties of aggregate and filler used in this study.

Technical Indices	Aggregate/Filler	Measure Value	Test Method	Criteria
Crushing value/%	CA	22.7	BS 812-110 [20]	Maximum of 35
	Basalt aggregate	10.6		
Los Angeles abrasion/%	CA	20.5	ASTM C131 [21]	Maximum of 40
	Basalt aggregate	11.4		
Flat and elongated particle content/%	CA	13.8	ASTM D4791 [22]	Maximum of 10
	Basalt aggregate	2.6		
Water absorption rate/%	CA	0.86	ASTM C127 [23]	None
	Basalt aggregate	0.32		
Density/g·cm^−3^	CA	2.348	ASTM C127 [23]	None
	Basalt aggregate	2.941		
	Limestone filler	2.723	ASTM C128 [24]	
Plasticity index	Limestone filler	Non-plastic	ASTM D4318 [25]	Non-plastic

**Table 2:** Basic properties of SBS modified asphalt.

Technical Indices	Measure Value	Test Method	Criteria
Penetration at 25 °C/0.1 mm	63.4	ASTM D5 [26]	50–70
Softening point/°C	72.0	ASTM D36 [27]	≥60
Ductility at 5 °C/cm	78.5	ASTM D113 [28]	≥30
Solubility in trichloroethylene/%	99.4	ASTM D2042 [29]	≥99
Flash point/°C	260	ASTM D92 [30]	≥230
Viscosity at 135 °C/Pa·s	2.16	ASTM D2170 [31]	≥3
Elastic recovery/%	94	ASTM D113 [28]	≥99
**After the Rolling Thin Film Oven Test (RTFOT)**			
Loss in weight/%	0.12	ASTM D1754 [32]	≥1.0
Retained penetration/%	64	ASTM D5 [26]	≥70
Ductility at 5 °C/cm	28	ASTM D113 [28]	≥20

**Table 3:** Marshall test results of stone mastic asphalt containing CA.

Index	SMA-13	CASMA-13					Criteria
		10%	20%	30%	40%	50%	
Optimum asphalt content/%	5.7	5.8	5.8	5.9	6.0	6.2	-
Density/g·cm^−3^	2.520	2.493	2.479	2.438	2.418	2.362	-
Air void/%	4.0	3.9	3.8	3.9	4.0	3.9	3.0–4.0
VFA/%	78.1	78.6	80.2	78.40	79.21	81.22	75–85
VMA/%	17.3	17.3	17.1	16.9	17.4	17.0	≥17
Stability/kN	8.65	7.39	7.36	7.28	7.13	6.57	≥6.0
Flow value/0.1 mm	20.8	21.0	23.5	22.8	25.61	28.37	20–50
Leakage loss/%	0.05	0.06	0.06	0.07	0.09	0.12	≥0.1
Scattering loss/%	3.5	5.5	5.8	7.3	11.5	13.2	≥15

**Table 4:** Test arrangement.

Test	Property	Material	Index	Test Method
Wheel rutting test	Deformation resistance	SMA-13 (0%), CASMA-13 (10%), CASMA-13 (20%), CASMA-13 (30%), CASMA-13 (40%), CASMA-13 (50%).	Dynamic stability	JTG E20-2011 T0719 [34]
Freeze-thaw indirect tensile test	Moisture susceptibility		Tensile strength ratio (TSR)	AASHTO T283 [29]
Bending beam test	Cracking resistance		Bending strength	JTG E20-2011 T0715 [34]
Thermophysical parameter test (hot disc method test)	Thermophysical performance		Conductivity	ASTM WK49591 [35]
			Diffusivity	
			Specific heat	
Anti-skid performance test	Anti-skid performance		British pendulum number (BPN)	ASTM E303 [36]
			Mean texture depth (MTD)	ASTM E965 [37]
Fatigue beam test	Fatigue performance		Failure cycles	CEN-EN 12697-24 [38]
Thermal insulation test	Thermal insulation performance		Temperature difference	Designed in this study

**Table 5:** Thermal parameters.

Item	SMA-13	AC-20C	AC-25C	CSM	Graded Gravel	Soil Base
Thermal conductivity/W m^−1^·K^−1^	1.302	1.070	1.000	1.560	1.200	1.560
Density/kg m^−3^	2520	2412	2333	2200	2000	1800
Specific heat/J kg^−1^ K^−1^	812.6	852.1	801.4	911.7	900.0	1040.0
Solar radiation absorption rate	0.90					
Emissivity coefficient	0.81					

**Table 6:** Creep parameters of surface course.

Time Hardening Creep Model	Asphalt Mixture Type	Temperature/°C	*A*	*n*	*m*	*R^2^*
${\overset{•}{\varepsilon }}_{cr}=Aq^{n}t^{m}$	SMA-13-0%	20	6.536 × 10^−11^	0.937	−0.592	0.9326
		30	3.325 × 10^−9^	0.862	−0.587	0.9459
		40	1.446 × 10^−8^	0.792	−0.577	0.9420
		50	1.390 × 10^−6^	0.414	−0.525	0.9244
		60	1.464 × 10^−5^	0.336	−0.502	0.9049
	CASMA-13-40%	20	5.029 × 10^−11^	0.909	−0.784	0.9855
		30	2.368 × 10^−9^	0.852	−0.778	0.9860
		40	3.267 × 10^−8^	0.767	−0.668	0.9693
		50	1.598 × 10^−6^	0.396	−0.637	0.9470
		60	1.698 × 10^−5^	0.341	−0.611	0.9523

**Table 7:** Test results of stone mastic asphalt.

Index	Temperature/°C	SMA−13	CASMA-13					Criterion
			10%	20%	30%	40%	50%	
Dynamic stability/pass·mm^−1^	60	5364	4652	4531	4181	3524	2036	≥3000
TSR/%	15	90.6	85.7	83.5	83.2	80.2	74.8	≥75
Failure bending strain/με	−10	3024	2751	2730	2698	2703	2583	≥2500
Thermal conductivity/W·m^−1^·K^−1^	20	1.302	1.035	0.831	0.716	0.611	0.570	-
Thermal diffusivity/10^−7^ m^2^·s^−1^		6.358	4.864	3.642	3.010	2.569	2.341	
Specific heat/J·kg^−1^·K^−1^		812	982	1093	1168	1269	1324	
BPN	25	73	70	72	70	71	72	≥45
Mean texture depth/mm	25	1.04	0.88	0.98	0.95	0.94	1.0 5	≥0.8

**Table 8:** Two-way ANOVA results for fatigue test.

Object	Factor	DF	*F*-Value	*p*-Value	Significance
Fatigue life	Stress ratio	3	2568.85	<0.001	***
	CA content	5	1.800	0.173	—

*** presents high significance; —presents no significance.

**Table 9:** Results of indoor thermal insulation performance test.

Index	SMA-13	CASMA-13				
		10%	20%	30%	40%	50%
Temperature of compound specimen surface/°C	68.5	68.9	69.1	69.6	70.7	70.9
Temperature of compound specimen interface/°C	66.1	63.1	61.4	60.5	60.3	59.7
Temperature of compound specimen bottom/°C	51.6	46.9	45.3	43.7	43.2	42.5
Temperature difference between surface and interface/°C	2.4	5.8	7.7	9.1	10.4	11.2
Temperature difference between surface and bottom/°C	16.9	22.0	23.8	25.9	27.5	28.4
